# Anxiety management in Australian general practice: an analysis of encounters from 2006 – 2016

**DOI:** 10.1186/s12875-023-02110-9

**Published:** 2023-08-04

**Authors:** Erin L. Parker, Michelle Banfield, Daniel B. Fassnacht, Christine B. Phillips, Christopher Harrison

**Affiliations:** 1 School of Medicine and Psychology, Australian National University, Canberra, ACT 2601 Australia; 2 Centre for Mental Health Research, Australian National University, Canberra, ACT Australia; 3 ALIVE National Centre for Mental Health Research Translation, Melbourne, VIC Australia; 4 College of Education, Psychology and Social Work, Flinders University, Adelaide, SA Australia; 5 School of Health, University of the Sunshine Coast, Sunshine Coast, QLD Australia; 6 Menzies Centre for Health Policy and Economics, School of Public Health, University of Sydney, Sydney, NSW Australia

**Keywords:** Anxiety, General practice, Primary care, Management

## Abstract

**Background:**

Anxiety disorders are highly prevalent mental health conditions managed predominantly by general practitioners (GPs). This study aimed to examine the management of anxiety by Australian GPs since the introduction of the Better Access to Psychiatrists, Psychologists and General Practitioners initiative in 2006.

**Methods:**

We conducted secondary analysis of Bettering the Evaluation and Care of Health data on GP encounters for anxiety from 2006 to 2016 (*N* = 28,784). We calculated point estimates and used multivariate logistic regression to explore the effect of GP and patient characteristics on rates and types of management.

**Results:**

The management rate of anxiety increased from 2.3% of GP encounters in 2006 to 3.2% in 2016. Over the 10-year period, increases were seen in referrals to psychologists (AOR = 1.09, 95%CI = 1.07–1.11, *p* < .0001) and selective serotonin / serotonin-noradrenalin reuptake inhibitors (AOR = 1.05, 95%CI = 1.03–1.06, *p* < .0001), and benzodiazepines decreased (AOR = 0.94, 95%CI = 0.92–0.95, *p* < .0001). Systematic differences in management were found for patient and GP characteristics, including high rates of benzodiazepines in certain groups.

**Conclusions:**

Anxiety is accounting for more of the GP workload, year on year. GP management of anxiety has become more closely aligned with practice guidelines since 2006. However, high rates of benzodiazepine prescribing in certain groups remains a concern. Further research is needed into GP treatment decision making for anxiety.

## Background

Anxiety disorders are highly prevalent mental health conditions that are predominantly managed in primary care [1]. Best practice treatment of anxiety involves stepped-care including both psychological and pharmacological interventions [2]. Choice of treatment should be based on severity of symptoms and functional impairment, co-occurring difficulties, consumer preferences, and previous treatment [2, 3], with psychological interventions generally recommended as first-line. However, pharmacological interventions are the most commonly provided treatment irrespective of anxiety severity, and less than half of people seeking help in primary care receive adequate evidence-based treatment [4].

In the past 20 years, there have been major reforms of mental health care in Australia. A key focus has been to improve primary mental health care by realising the role GPs have to play. In particular, the Better Access to Psychiatrists, Psychologists and General Practitioners (Better Access) initiative introduced in 2006 [5], allows consumers to access rebates for evidence-based psychological services provided by mental health professionals (mainly psychologists) from Medicare, the government-funded universal health insurance. In this system, GPs act as both primary providers of treatment and a gateway to specialist mental health care, facilitating access to subsidised psychological services through the creation of a Mental Health Treatment Plan, which involves preliminary assessment, identification of goals, and exploration of treatment options [5].

Previous research has investigated the impact of these reforms on mental health care, though little has focussed on anxiety specifically; several studies have analysed rates of access and management for various mental health conditions such as suicide-related contacts [6], general psychological problems [7], and serious mental illness [8, 9]. A 2012 study found that following the introduction of Better Access, rates of depression management within primary care increased, as did referrals to psychologists [10]. However, absolute rates of specialist referral for mental health conditions remained relatively low, with referral to psychologists (the most common type) occurring in less than 10 per cent of mental-health related encounters. Reports on general practice activity in subsequent years have displayed a continued increase in referral rates [11]. The program has now achieved wide reach; by 2021, one in 20 Australians had received at least one psychological service through Better Access [12]. Over 70% of diagnoses for people treated under Better Access are anxiety disorders (alone or with other conditions) [12], though trends in treatment are not available.

Anxiety disorders tend to be chronic if inadequately treated, resulting in substantial impairment for the individual and high economic costs [13, 14]. There has been ample discussion about the effective treatment of anxiety over the last two decades and updated clinical practice guidelines have been published in Australia and internationally [2, 3, 15]. In particular, as the overuse and limitations of benzodiazepines for anxiety are well documented (e.g., [16]), practice guidelines emphasise a move away from these medications. Formal monitoring and restrictions on benzodiazepines have also been introduced, and the use of these medications generally has been declining [17]. However, little research has investigated the rates of treatment for anxiety within Australian primary care, and in particular, current prescribing practices.

The current study aimed to address these gaps by examining general practice encounters for anxiety in the 10-year period following the introduction of the Better Access program. Further, we explored the effect of GP and patient characteristics on the likelihood of different treatments being used to manage anxiety problems.

## Method

The Bettering the Evaluation and Care of Health (BEACH) program was a continuous, national study of general practice activity conducted over 18 years, from April 1998 to June 2016. Each year, a different random sample of 1,000 currently practicing GPs provided details on 100 consecutive consenting patient encounters. For each visit, GPs recorded the reason for the visit, the problem managed during the encounter, any treatment delivered (e.g., clinical treatments, prescriptions provided), and any referrals to other health professionals. The BEACH dataset represents the most current and comprehensive record of GP activity in Australia [11, 18]. In total, the database includes approximately 1.78 million patient encounters, recorded by 11,000 GPs. Further details about the methods used in the BEACH program have been published previously [19].

The BEACH study has approval from both the Human Research Ethics Committee of the University of Sydney (Protocol 2012/130) and from the Ethics Committee of the Australian Institute of Health and Welfare for the years they collaborated (2006–11). Extraction and analysis of BEACH data for the current study was approved by the Australian National University Human Research Ethics Committee (Protocol: 2020/542).

### Participants and measures

At each encounter, GPs could record up to four problems managed. The reason for encounter, problems managed, and non-pharmacological treatments were coded by trained research staff according to the International Classification of Primary Care Version 2 PLUS (ICPC-2 PLUS, [20]). Data were then automatically classified to ICPC-2 [21]. Pharmacological treatments were coded according to the Anatomical Therapeutic Chemical (ATC) classification system [22]. For the current analyses, we defined anxiety using the following codes ‘P01’ (feeling anxious), ‘P74’ (anxiety disorder), or ‘P76018’ (anxiety with depression’).

In addition, we extracted data on the following patient variables: sex, age, Commonwealth Health Care Card status (i.e., whether or not a person holds a Government healthcare concession card, which are held by those receiving disability, unemployment, student, carer, aged pension, or other Government income supplements), language background (i.e., language spoken at home; English speaking vs. non-English speaking), and Aboriginal or Torres Strait Islander status (i.e., whether or not a person is Aboriginal or Torres Strait Islander). We used patient residential postcode to define relative socioeconomic advantage according to the Socio-Economic Indexes for Areas (SEIFA) Index of Relative Socio-economic Advantage and Disadvantage (IRSAD, [23]). We compared patients from “most advantaged” (top five IRSAD deciles) areas to those from “most disadvantaged” (bottom five IRSAD deciles) areas in our analyses. Data were also extracted on the following GP characteristics: sex, age, practice size, and practice location (major cities vs. inner regional vs. outer regional/remote) according to the Australian Statistical Geography Standard (ASGS, [24]).

### Statistical analysis

All point estimates were calculated as proportions for ease of interpretation, that is, outcomes that could happen more than once per instance (e.g., medications used in treatment) were only counted once. The BEACH study has a single cluster design with each GP having a cluster of 100 patient encounters around them. We used the surveymeans procedures in SAS v9.4 [25] to produce robust 95% confidence intervals as this procedure adjusts for any intracluster correlation. For our descriptive analyses, we judged two point estimates as being significantly different by non-overlapping 95% confidence intervals. This method is far more conservative than the usual alpha of 0.05 [26].

We also performed several multivariate logistic regression analyses to identify the independent predictors of anxiety being managed at an encounter as well as independent predictors of certain treatments being used to manage anxiety (selective serotonin reuptake inhibitors [SSRIs]/serotonin noradrenalin reuptake inhibitors [SNRI], benzodiazepines, counselling/advice/education, referral to a psychologist). We explored these treatments specifically as they are the most commonly used by GPs and the most relevant in terms of clinical practice guidelines. We created a combined outcome category for counselling, advice, or education (CAE) provided by the GP (which included, for example, psychoeducation about anxiety, advice about lifestyle factors, supportive counselling, counselling about medication use). We also combined SSRI and SNRI medications into one outcome category due to both being recommended first-line agents for anxiety problems commonly presenting in primary care [15, 27].

## Results

### Management rate of anxiety 2006 – 2016

**Fig. 1:**
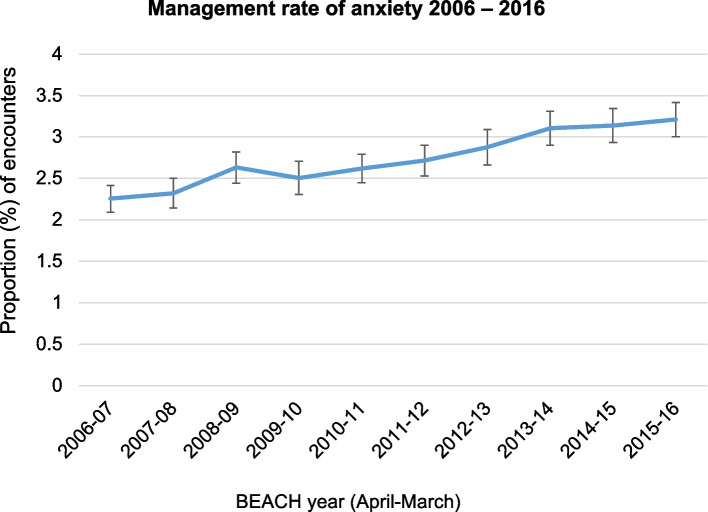
Proportion of GP encounters where anxiety was managed by year 2006–16 (error bars = 95% CIs)

Over the 10-year study period, 9,721 GPs recorded 972,100 encounters with patients. A total of 28,849 anxiety problems were recorded at 28,784 encounters, accounting for 3.0% of general practice encounters (95% CI 2.9 – 3.0). Figure 1 shows the management rate of anxiety from 2006 – 2016, measured as the proportion of all encounters per year. There was an almost 40% increase in the management rate of anxiety, from 2.3% (95%CI = 2.1–2.4) in 2006–07 to 3.2% (95%CI = 3.0–3.4) in 2015–16.

Most anxiety problems (71.7%) were recorded using the codes ‘P01’ (feeling anxious) or ‘P74’ (anxiety disorder), and 28.3% were recorded under mixed anxiety/depression (‘P76018’). New anxiety problems (*N* = 5,023) accounted for 17.4% of total anxiety problems, and 62.4% (*N* = 18,014) were recorded as an existing condition. Data were missing for this variable in the remaining encounters.

Table 1 reports the management rate of anxiety over the 2006 – 2016 period by patient and GP characteristics. Patients were more likely to have anxiety managed at an encounter if they were female, socioeconomically advantaged, not Aboriginal or Torres Strait Islander, from an English speaking background, and a Commonwealth Health Care Card holder. Patient age was also associated with likelihood of having anxiety managed at an encounter, with the highest proportion found in 25–39 year olds, followed by 40–59 year olds. Patients under 15 years of age were the least likely to have anxiety managed. GPs were more likely to manage anxiety if they were female, older, and working in less remote practice locations.

**Table 1:** Management rate of anxiety in general practice 2006 – 2016 by patient and GP characteristics

Variable	Total sample (*N* = 972100)	Anxiety encounters (*N* = *2784*)	Proportion (95% CI)	Adjusted OR (95%CI)
**Patient sex** *(missing)*	*(8522)*	*(235)*		*p* < .0001
Male	391152	9032	2.31% (2.24–2.38)	Ref group
Female	572426	19517	3.41% (3.34–3.48)	1.31 (1.27–1.35)
**Patient age** *(missing)*	*(19222)*	*(730)*		*p* < .0001
0–14 years	110864	705	0.64% (0.58–0.69)	0.48 (0.43–0.54)
15–24 years	81201	2792	3.44% (3.27–3.61)	2.56 (2.37–2.77)
25–39 years	148287	6406	4.32% (4.18–4.46)	3.33 (3.11–3.57)
40–59 years	254450	10201	4.01% (3.90–4.12)	3.02 (2.83–3.22)
60–80 years	259108	6157	2.38% (2.30–2.45)	1.41 (1.33–1.49)
80 + years	98968	1793	1.81% (1.72–1.91)	Ref group
**Socioeconomic advantage** *(missing)*	*(22692)*	*(607)*		*p* < .0001
Most advantaged	573803	17396	3.03% (2.96–3.10)	1.08 (1.04–1.12)
Most disadvantaged	375605	10781	2.87% (2.79–2.95)	Ref group
**Indigenous status**^**a**^ *(missing)*	*(95622)*	*(2561)*		*p* = .014
Indigenous	14791	427	2.89% (2.54–3.23)	0.86 (0.76–0.97)
Non-Indigenous	861687	25796	2.99% (2.93–3.06)	Ref group
**Language background** *(missing)*	*(95865)*	*(2575)*		*p* < .0001
Non-English speaking	74672	1642	2.20% (2.05–2.34)	0.65 (0.6–0.70)
English speaking	801563	24567	3.06% (3.00–3.13)	Ref group
**Commonwealth HCC** *(missing)*	*(80058)*	*(2202)*		*p* < .0001
Yes	396992	13450	3.39% (3.30–3.48)	1.73 (1.67–1.80)
No	495050	13132	2.65% (2.59–2.72)	Ref group
**Practice location** *(missing)*	*(1400)*	*(29)*		*p* < .0001
Major city	687500	21046	3.06% (2.99–3.13)	1.34 (1.24–1.45)
Inner regional	187800	5515	2.94% (2.81–3.06)	1.27 (1.17–1.38)
Outer regional/remote	95400	2194	2.30% (2.15–2.45)	Ref group
**Practice size** *(missing)*	*(18900)*	*(533)*		*p* = .991
Solo	48600	2921	2.82% (2.60–3.04)	Ref group
2–4 GPs	127600	8177	2.85% (2.74–2.96)	0.99 (0.90–1.09)
5–9 GPs	108200	10962	3.00% (2.91–3.09)	1.00 (0.91–1.10)
10–14 GPs	28700	4307	3.16% (2.99–3.32)	1.01 (0.91–1.12)
15 + GPs	10000	1884	3.11% (2.87–3.35)	1.00 (0.89–1.13)
**GP sex** *(missing)*	*(0)*	*(0)*		*p* < .0001
Male	583200	15383	2.64% (2.56–2.71)	Ref group
Female	388900	13401	3.45% (3.35–3.54)	1.23 (1.17–1.28)
**GP age** *(missing)*	*(6400)*	*(122)*		*p* < .0001
Less than 45 years	250500	6883	2.75% (2.65–2.85)	Ref group
45–59 years	473400	14404	3.04% (2.96–3.13)	1.19 (1.13–1.25)
60 years or older	241800	7375	3.05% (2.92–3.18)	1.25 (1.17–1.33)
**Data collection year**	N/A	N/A	N/A	*p* < .0001 1.05 (1.04–1.05)

*OR* odds ratio, *HCC* health care card, *GP* general practitioner

^a^Aboriginal and/or Torres Strait Islander (patient self-report)

### Management strategies used

**Fig. 2:**
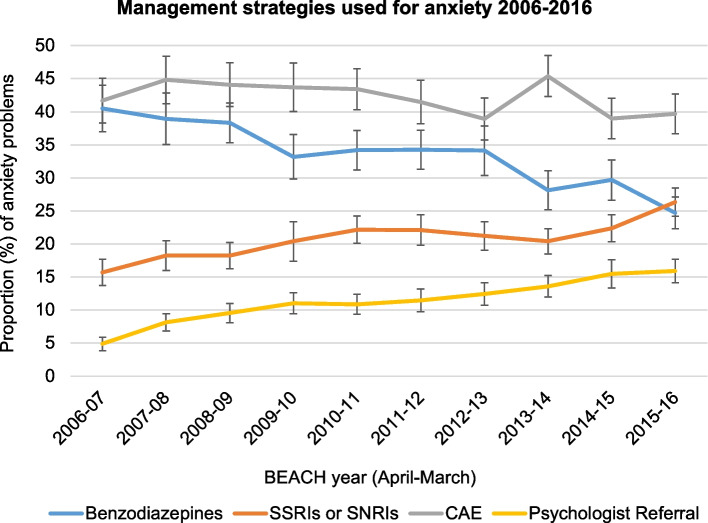
Proportion of anxiety problems where management strategy was used by year 2006–16 (error bars = 95% CIs)

A summary of management strategies used for anxiety problems is reported in Table 2. GPs were significantly more likely to manage anxiety with psychotropic medications than any other approach. There was a significant, linear reduction in the proportion of anxiety problems managed with benzodiazepines across the 10 years (see Fig. 2, Table 4), reducing from 40.5% (95%CI = 37.0 – 44.0) in 2006 to 24.7% (95%CI = 22.3 – 27.1) in 2016. Additionally, the use of SSRI/SNRI medications increased year on year, from 15.7% (95%CI = 13.7 – 17.7) in 2006 to 26.3% (95%CI = 24.2 – 28.5) in 2016.

**Table 2:** Management strategies used for anxiety 2006—2016

Management Strategy	Anxiety problems managed with strategy least once (*N* = 28849)	Proportion of anxiety problems managed with strategy at least once (95% CI)
**Psychotropic medication**	15238	52.8% (52.0–53.7)
Benzodiazepine	8664	30.0% (29.2–30.9)
SSRI or SNRI	6084	21.1% (20.5–21.7)
**Counselling / advice / education**	12601	43.7% (42.8–44.6)
**Referral**	4900	17.0% (16.5–17.5)
Psychologist	3522	12.2% (11.7–12.7)
Psychiatrist	543	1.9% (1.7–2.0)
**Pathology**	1312	4.5% (4.3–4.8)
**Imaging**	138	0.5% (0.4–0.6)

More than one management strategy could be recorded for each encounter, so proportions add to more than 100%

*SSRI* selective serotonin reuptake inhibitor, *SNRI* serotonin noradrenalin reuptake inhibitor

The most common single strategy used by GPs was counselling, advice, or education (CAE), which occurred at higher rates than prescriptions of benzodiazepines and SSRI or SNRI medications. Referrals were given for 17% of anxiety problems, and were most commonly to psychologists (12.2% of anxiety problems). The rate of psychologist referral increased substantially over the period studied, from 4.9% (95%CI = 3.8 – 5.9) in 2006 to 15.9% (14.2 – 17.7) in 2016.

Unadjusted proportions for anxiety management with benzodiazepines, SSRIs/SNRIs, CAE, and referral to a psychologist by year and patient and GP characteristics are reported in Table 3 (patient characteristics) and Table 4 (GP characteristics and year), along with multivariate logistic regression results demonstrating the independent effect of each variable on management rate. All variables except Aboriginal or Torres Strait Islander status significantly predicted likelihood at least one of the four strategies being used to manage anxiety. Notable results are discussed in text below.

**Table 3:** Unadjusted proportions and adjusted odds ratios (multivariate logistic regression) of anxiety problems managed with different strategies, by patient characteristics

	**Benzodiazepines**		**SSRIs or SNRIs**		**CAE**		**Psychologist Referral**	
**Variable**	**Proportion** **(95%CI)**	**Adjusted OR** **(95%CI)**	**Proportion** **(95% CI)**	**Adjusted OR** **(95%CI)**	**Proportion** **(95% CI)**	**Adjusted OR** **(95%CI)**	**Proportion** **(95% CI)**	**Adjusted OR** **(95%CI)**
**Patient sex**		*p* < .0001		*p* < .0001		*p* = .729		*p* = .055
Male	34.3% (32.8–35.7)	Ref group	39.5% (38.2–40.8)	Ref group	11.8% (11.1–12.6)	Ref group	19.9% (18.9–20.8)	Ref group
Female	28.1% (27.2–29.0)	0.81 (0.75–0.87)	45.6% (44.7–46.6)	1.21 (1.13–1.29)	12.4% (11.9–12.9)	1.02 (0.92–1.12)	21.6% (20.9–22.3)	1.08 (1.00–1.17)
**Patient age**		*p* < .0001		*p* < .0001		*p* < .0001		*p* < .0001
0–14 years	0.1% (-0.1–0.4)	0.01 (< 0.001–0.04)	39.5% (35.7–43.4)	0.76 (0.60–0.96)	39.1% (35.3–42.9)	26.06 (15.9–42.73)	6.5% (4.7–8.3)	0.37 (0.25–0.55)
15–24 years	10.1% (8.9–11.4)	0.25 (0.20–0.30)	50.2% (48.0–52.3)	1.38 (1.17–1.63)	23.1% (21.4–24.9)	14.14 (8.86–22.55)	26.4% (24.6–28.1)	1.90 (1.56–2.31)
25–39 years	26.3% (24.8–27.8)	0.72 (0.62–0.84)	45.3% (43.8–46.9)	1.20 (1.05–1.39)	16.7% (15.7–17.7)	10.00 (6.29–15.90)	24.5% (23.3–25.6)	1.78 (1.49–2.14)
40–59 years	32.2% (30.9–33.4)	0.87 (0.75–0.99)	43.9% (42.7–45.2)	1.22 (1.06–1.39)	10.5% (9.8–11.1)	6.73 (4.25–10.65)	22.2% (21.3–23.1)	1.55 (1.30–1.85)
60–80 years	39.1% (37.8–40.5)	0.88 (0.77–1.01)	41.0% (39.5–42.4)	1.12 (0.99–1.28)	5.0% (4.4–5.5)	3.43 (2.15–5.49)	17.0% (16.0–18.0)	1.30 (1.08–1.56)
80 + years	44.0% (41.5–46.4)	Ref group	37.9% (35.5–40.4)	Ref group	1.5% (0.9–2.1)	Ref group	13.0% (11.4–14.6)	Ref group
**Socioeconomic status**		*p* < .0001		*p* = .095		*p* = .317		*p* = .380
Most advantaged	27.0% (26.0–27.9)	0.85 (0.78–0.92)	45.6% (44.5–46.7)	1.07 (0.99–1.16)	13.3% (12.7–13.9)	1.06 (0.95–1.18)	21.3% (20.5–22.0)	1.04 (0.95–1.13)
Most disadvantaged	35.4% (34.0–36.7)	Ref group	40.6% (39.3–41.9)	Ref group	10.4% (9.8–11.1)	Ref group	20.6% (19.7–21.5)	Ref group
**Indigenous status**^**a**^		*p* = 0.227		*p* = .179		*p* = .529		*p* = .445
Indigenous	37.9% (32.3–43.5)	1.18 (0.90–1.54)	35.1% (29.6–40.6)	0.83 (0.62–1.09)	12.3% (8.9–15.8)	1.12 (0.78–1.61)	21.9% (17.7–26.0)	1.11 (0.85–1.46)
Non-Indigenous	30.5% (29.5–31.4)	Ref group	43.9% (43.0–44.9)	Ref group	12.1% (11.6–12.6)	Ref group	20.8% (20.2–21.5)	Ref group
**Language background**		*p* < .0001		*p* = .0005		*p* = .0001		*p* = .003
Non-English speaking	28.2% (25.5–30.8)	0.73 (0.63–0.85)	51.6% (48.7–54.6)	1.28 (1.11–1.47)	7.9% (6.5–9.3)	0.64 (0.51–0.80)	15.4% (13.5–17.3)	0.77 (0.65–0.92)
English speaking	30.8% (29.8–31.7)	Ref group	43.3% (42.3–44.2)	Ref group	12.3% (11.8–12.9)	Ref group	21.2% (20.6–21.9)	Ref group
**Commonwealth HCC**		*p* < .0001		*p* < .0001		*p* < .0001		*p* < .0001
Yes	42.2% (40.9–43.5)	2.50 (2.31–2.72)	39.1% (37.9–40.3)	0.80 (0.75–0.86)	8.0% (7.5–8.6)	0.78 (0.70–0.86)	17.2% (16.4–17.9)	0.66 (0.61–0.71)
No	18.4% (17.6–19.2)	Ref group	48.5% (47.4–49.7)	Ref group	16.3% (15.6–17.0)	Ref group	24.9% (24.0–25.8)	Ref group
**Type of anxiety**		*p* < .0001		*p* < .0001		*p* < .0001		*p* < .0001
New problem	16.3% (15.2–17.4)	0.43 (0.39–0.48)	55.6% (54.0–57.2)	1.85 (1.71–2.01)	20.8% (19.6–22.1)	2.05 (1.85–2.27)	16.9% (15.8–18.1)	0.62 (0.56–0.68)
Existing problem	34.6% (33.4–35.7)	Ref group	40.6% (39.5–41.7)	Ref group	10.0% (9.5–10.5)	Ref group	23.6% (22.8–24.3)	Ref group

*OR* Odds ratio, *SSRIs* Selective serotonin reuptake inhibitors, *SNRIs* Serotonin noradrenalin reuptake inhibitors, *HCC* Health care card, *CAE* Counselling / advice / education

^a^Aboriginal and/or Torres Strait Islander (patient self-report)

**Table 4:** Unadjusted proportions and adjusted odds ratios (multivariate logistic regression) of anxiety problems managed with different strategies, by GP characteristics

	**Benzodiazepines**		**SSRIs or SNRIs**		**CAE**		**Psychologist Referral**	
**Variable**	**Proportion** **(95%CI)**	**Adjusted OR** **(95%CI)**	**Proportion** **(95% CI)**	**Adjusted OR** **(95%CI)**	**Proportion** **(95% CI)**	**Adjusted OR** **(95%CI)**	**Proportion** **(95% CI)**	**Adjusted OR** **(95%CI)**
**Practice location**		*p* = .641		*p* = .003		*p* = .035		*p* = .004
Major city	29.2% (28.2–30.2)	1.04 (0.89–1.23)	45.2% (44.1–46.2)	1.26 (1.07–1.48)	12.8% (12.3–13.4)	1.19 (0.97–1.47)	20.6% (19.8–21.3)	0.90 (0.77–1.05)
Inner regional	32.0% (30.1–34.0)	0.99 (0.83–1.18)	39.8% (37.9–41.7)	1.09 (0.92–1.30)	10.7% (9.8–11.7)	1.02 (0.81–1.28)	23.2% (21.8–24.6)	1.08 (0.91–1.28)
Outer regional/remote	32.7% (29.8–35.6)	Ref group	39.2% (36.2–42.3)	Ref group	9.9% (8.5–11.4)	Ref group	21.3% (19.3–23.4)	Ref group
**Practice size**		*p* = .315		*p* = .658		*p* = .017		*p* = .001
Solo GP	42.0% (38.1–46.0)	Ref group	39.3% (35.8–42.9)	Ref group	6.5% (5.4–7.6)	Ref group	14.4% (12.7–16.1)	Ref group
2–4 GPs	31.2% (29.5–32.9)	0.87 (0.71–1.07)	44.4% (42.7–46.1)	1.07 (0.88–1.29)	11.2% (10.4–12.0)	1.33 (1.05–1.70)	20.8% (19.7–21.9)	1.46 (1.21–1.76)
5–9 GPs	27.9% (26.7–29.1)	0.82 (0.67–1.00)	43.9% (42.6–45.3)	1.08 (0.90–1.30)	13.5% (12.7–14.3)	1.48 (1.17–1.87)	21.8% (20.9–22.7)	1.40 (1.17–1.68)
10–14 GPs	26.2% (24.3–28.0)	0.82 (0.66–1.02)	45.7% (43.4–47.9)	1.15 (0.93–1.40)	14.0% (12.7–15.2)	1.39 (1.08–1.79)	22.9% (21.4–24.5)	1.36 (1.11–1.67)
15 + GPs	28.0% (24.4–31.5)	0.80 (0.62–1.04)	42.5% (38.8–46.2)	1.02 (0.80–1.31)	13.5% (11.5–15.4)	1.30 (0.97–1.73)	23.9% (21.3–26.6)	1.56 (1.23–1.97)
**GP sex**		*p* < .0001		*p* < .0001		*p* < .0001		*p* < .0001
Male	38.0% (36.7–39.3)	Ref group	39.1% (37.9–40.4)	Ref group	9.7% (9.1–10.3)	Ref group	18.6% (17.8–19.4)	Ref group
Female	20.8% (19.9–21.8)	0.56 (0.51–0.62)	48.9% (47.6–50.2)	1.29 (1.18–1.41)	15.1% (14.4–15.8)	1.30 (1.16–1.44)	23.9% (23.0–24.8)	1.22 (1.11–1.33)
**GP age**		*p* < .0001		*p* < .0001		*p* < .0001		*p* < .0001
< 45 years	22.1% (20.9–23.3)	Ref group	45.5% (43.7–47.2)	Ref group	16.4% (15.4–17.4)	Ref group	26.1% (24.9–27.3)	Ref group
45–59 years	28.4% (27.3–29.6)	1.18 (1.06–1.31)	45.8% (44.5–47.1)	1.08 (0.97–1.19)	12.4% (11.7–13.0)	0.89 (0.80–1.00)	20.9% (20.1–21.7)	0.82 (0.74–0.90)
60 years +	40.6% (38.6–42.7)	1.65 (1.44–1.88)	37.8% (36.0–39.6)	0.82 (0.72–0.94)	8.0% (7.3–8.8)	0.68 (0.58–0.81)	16.8% (15.6–18.0)	0.70 (0.62–0.80)
**Data collection year**	n/a	*p* < .0001 0.94 (0.92–0.95)		*p* = .009 0.98 (0.96–1.00)		*p* < .0001 1.09 (1.07–1.11)	n/a	*p* < .0001 1.05 (1.03–1.06)

*OR* Odds ratio, *SSRIs* Selective serotonin reuptake inhibitors, *SNRIs* Serotonin noradrenalin reuptake inhibitors, *CAE* Counselling / advice / education, *GP* General practitioner

#### Effect of patient characteristics

Existing anxiety problems were twice as likely to be managed with benzodiazepines and 61% more likely to be managed with SSRIs/SNRIs than new anxiety problems. The opposite pattern was seen for psychologist referrals and CAE from the GP, with existing anxiety being half as likely as new anxiety to be managed with either of these strategies.

Older patients were more likely to receive benzodiazepines and less likely to receive other management strategies than younger patients (with the exception of people under the age of 15, who were the least likely to receive SSRIs and CAE). Patients aged less than 15 years were the most likely to receive a referral to a psychologist. Patient sex was associated with likelihood of receiving benzodiazepines and CAE, with male patients 23% more likely to receive benzodiazepines, and female patients 21% more likely to receive CAE.

Patients from a non-English speaking background were approximately three quarters as likely to receive management with benzodiazepines or SSRIs/SNRIs, and two thirds as likely to receive a psychologist referral than patients from an English-speaking background. However, they were 28% more likely to receive CAE from their GP.

Holding a HCC was associated with two and a half times the likelihood of receiving a benzodiazepine, and a decreased likelihood of anxiety being managed with any of the other three strategies. Similarly, likelihood of receiving benzodiazepines was higher for the most socioeconomically disadvantaged patients.

#### Effect of GP characteristics

Female GPs were almost half as likely to manage anxiety with benzodiazepines, 22% more likely to use SSRIs/SNRIs, 29% more likely to provide CAE, and 30% more likely to refer to a psychologist than their male peers. Older GPs were also more likely to manage anxiety using benzodiazepines and less likely to use other management strategies. Compared with those aged under 45 years, GPs over 60 years old were 65% more likely to use benzodiazepines, and significantly less likely to use any of the other strategies to manage anxiety problems.

## Discussion

### Management rate of anxiety 2006 – 2016

Over the period analysed, this study demonstrated that anxiety was accounting for a larger proportion of GP workload year by year. In the absence of any other significant health reforms that would impact the management rate of anxiety, it is reasonable to assume that the observed linear increase would have continued from 2016 to 2023. The drivers of this increase are likely multifaceted, including changes to help-seeking, access, and prevalence. The introduction of Better Access was intended to lower barriers to accessing psychologists through the creation of Government rebates for these services, which consumers access by visiting their GP for initial assessment and referral under a Mental Health Treatment Plan [10], As such, consumers have been directed to seek care from a GP as the “first port of call” for managing anxiety and other mental health conditions. The background prevalence of anxiety may also have increased over the period studied, though this is difficult to determine due to the lack of large scale studies on the prevalence of mental health conditions in Australia during the study period [18]. As a result of the COVID-19 pandemic, rates of general practice encounters for anxiety may have increased further since 2020 due to increased onset of new anxiety, exacerbation of existing conditions, or increased help-seeking due to stressors and a lack of alternative coping strategies [28, 29].

### Management strategies used

Psychotropic medications were the most common treatment category, but GPs managed anxiety problems with CAE more often than either of the most common medications used (benzodiazepines and SSRI/SNRIs). Rates of referral to psychologists were relatively low. Despite the lowering of financial barriers for psychologists through Government rebates, psychological treatment remains expensive, and GPs are limited by a shortage of psychologists for private referral. However, referrals to psychologists tripled from 2006 to 2016 following the introduction of Better Access, suggesting the introduction of rebates facilitated increased access to private psychology as intended. Non-pharmacological strategies were also particularly more common for new anxiety problems, suggesting alignment with practice guidelines that recommend psychological interventions as first line for anxiety [2, 3].

Although approximately one third of anxiety problems were managed with a benzodiazepine, the use of these medications reduced substantially over the 10 years. Issues with benzodiazepines have been well documented, and reducing their use has been the focus of a significant amount of education and policy. New Australian guidelines for the use of benzodiazepines in general practice were released in 2015, including specific recommendations for managing anxiety [30] in line with international guidance [3], which may have let to further decreases in benzodiazepine prescribing for anxiety from 2016 to the current time.

### Effect of patient and GP characteristics

There was a complex interaction between sex of GPs and patients. Female patients were more likely than males to have anxiety managed, consistent with the higher prevalence of anxiety in women [14, 31] and greater likelihood of seeking help for mental health problems [32]. However, female patients are also more likely to see female GPs [33], who were more likely to manage anxiety in our sample.

Female patients were more likely to receive CAE and less likely to receive benzodiazepines than male patients. This again may be explained, in part, by higher likelihood of seeing a female GP, who were much more likely to manage anxiety with CAE and much less likely to use benzodiazepines. Older and male GPs were more likely to manage anxiety with benzodiazepines, and less likely to use other management strategies than younger and female GPs. This is consistent with patterns of management for other conditions, which has shown female GPs are more likely to provide preventative care, referrals, and counselling [34]. Overall, the pattern also suggests younger and female GPs manage anxiety in ways more closely aligned to clinical practice guidelines, though this may be influenced by the patients they see.

Patients aged 25–59 had the highest rates of anxiety management, and rates were lowest in those under 15 years and over 80 years old. Young and middle-aged adults have the highest prevalence of anxiety, which typically peaks in middle age and decreases in older age [14, 31]. Further, the age of onset for anxiety disorders most seen in primary care (generalised anxiety disorder and panic disorder) is early adulthood [10], though long delays in help-seeking mean people may not present for treatment until a decade after symptom onset [35].

In older patient groups, there was a substantially increased likelihood of anxiety being managed with benzodiazepines and decreased likelihood of being referred to a psychologist. Benzodiazepine use is known to increase with age, with high rates of chronic use in the elderly [36, 37]. GPs report reluctance to cease benzodiazepines in these groups due to concerns about withdrawal and resistance from patients [38, 39]. People in older age groups are also more likely to be receiving management for an existing anxiety problem and may previously have received other treatments. However, the high rate of benzodiazepines for anxiety in the elderly is concerning; people over 60 years have a much higher risk of adverse effects relating to falls and confusion [30]. Patients under 15 years received very low rates of medication for anxiety, and relatively high rates of referral to a psychologist (26 times the likelihood of those aged over 80 years). These findings are consistent with recommendations for anxiety in children that emphasise psychological treatment as first-line [40].

The influence of socioeconomics was mixed. Anxiety was managed more often in patients from socioeconomically advantaged areas compared with disadvantaged areas, but HCC holders were 1.75 times more likely to have anxiety managed than non-card holders. It may be the case that people with a HCC, who are older, have a disability, or are low-income earners, experience higher rates of anxiety due to psychosocial stressors, financial disadvantage, and chronic illness. Regarding type of management, HCC holders were also almost three times as likely to receive benzodiazepines as people without a healthcare card, but significantly less likely to receive other management strategies. This indicates that HCC holders receive less preventative care for their anxiety, mirroring the management they receive for other health problems [41].

People from a non-English speaking background were far less likely to have anxiety managed at an encounter. Language background, although not a measure of ethnicity, is strongly predictive of being a member of a minority racial group [42]. Stigma, perceived barriers to mental healthcare, and cultural differences in recognition and help-seeking practices may mean people visit their GP fewer times for anxiety management [42, 43]. Systematic differences in the way anxiety was managed were also found for language background. People from a non-English speaking background were very unlikely to be referred to a psychologist and less likely to receive medication for anxiety than their English speaking counterparts. Language barriers and a lack of culturally competent psychologists may mean GPs are less likely to refer these groups for psychological therapy. Furthermore, stigma may result in reluctance, or perceived reluctance by the GP, to receive treatment for a mental health problem [43]. The treatments available in a migrant group’s country of origin are also likely to impact expectations about treatment in Australia (e.g., [44]). Finally, it may also be the case these findings are related to lower overall rates of consultations for anxiety.

Aboriginal and Torres Strait Islander people were less likely to have anxiety managed than non-Indigenous people. However, Aboriginal and Torres Strait Islander people are known to have higher rates of mental health difficulties than non-Indigenous people [45], and it may therefore be expected that they have anxiety managed at higher rates.

### Strengths and limitations

BEACH represents the most comprehensive and current dataset available on GP encounters within Australia. Unfortunately, the BEACH program was defunded and data are no longer being collected, so we were unable to track the treatment of anxiety in general practice beyond 2016 to describe the current management practices. However, examining 10 years of data provides information about trends that can be extrapolated to the current time. A limitation of the BEACH data is that they are cross sectional, meaning we were not able to determine whether a patient had received other management strategies at a previous encounter, or would at a future encounter. People could also receive more than one treatment at an encounter, and exploring the number of problems being managed with a single strategy (e.g., only benzodiazepines) was beyond the scope of this study.

Anxiety was recorded using two codes, one of which refers to a diagnosis of an anxiety disorder (P74), and another (P01) which includes the term “Anxiety”. We also included anxiety recorded under a third code (P76018) which refers to anxiety with depression. The use of multiple codes in the current study may have resulted in the inclusion of sub-clinical anxiety presentations, leading to an overestimation of the prevalence of anxiety disorder. Furthermore, the ICPC codes used by BEACH do not contain information about anxiety severity, so we are unable to determine any differences in management across this variable.

The results for CAE were also difficult to interpret, as the data are not fine-grained enough to determine exactly what GPs are providing when they record this as a management strategy. For instance, it may involve psychoeducation about anxiety, information about medications, advice about lifestyle factors, or brief psychological interventions. Furthermore, unlike medication and referrals, deciding whether counselling, advice, or education has been provided depends on interpretation from the GP, and the same strategy is likely to be recorded differently across different practitioners. Although we combined multiple categories of advice, counselling, and education into one outcome in our study, it was not meaningful to examine them separately due to the factors above.

There may also have been variations in estimations introduced by the nature of the data. Each anxiety problem managed did not represent an individual patient. Patients are likely to have had their anxiety managed multiple times across the 10 years and could have received the same or different strategies at each encounter. Benzodiazepines can only be prescribed in small amounts for a limited period under the Australian Pharmaceutical Benefits Scheme and will therefore require more GP encounters than a patient being treated with antidepressant medication (which can be provided for a period of six months under the Pharmaceutical Benefits Scheme) or psychologist referral. This is also reflected in high rates of CAE, which is a strategy that can be provided at every encounter unlike medications and psychologist referral.

### Future research and clinical implications

Our results suggest that anxiety is accounting for an increasing proportion of GP workload. We can expect that if anything, fallout from the COVID-19 pandemic will result in a larger increase in anxiety presentations than the linear pattern seen over the last few years. While milder anxiety presentations may resolve spontaneously, anxiety disorders tend to be chronic if insufficiently treated and it is important that appropriate management is provided [14].

High rates of benzodiazepine use in certain groups, particularly the elderly, are a concern. While benzodiazepines do have a place in the treatment of anxiety, practitioners should continue to reserve these medications for short-term use and in conjunction with other evidence-based treatments (e.g., during initiation of an SSRI/SNRI). The limitations in terms of effectiveness and the possibility for them to prolong anxiety disorders should be discussed with patients as well as tolerance/dependence issues to allow informed treatment decision making. Emphasising psychological treatments and reducing benzodiazepine use for anxiety in the elderly should be a priority.

Further research should explore GP treatment decision-making for anxiety to examine drivers behind the use of different management options, and differences across patient populations. Future research should also seek to understand consumer priorities for anxiety treatment, as there is some indication that consumers prefer psychological treatments over pharmacotherapy for common mental health problems [45, 46].

Although financial barriers have been lowered for treatment with a psychologist, it is acknowledged that this remains inaccessible for many [12]. Where referral to a psychologist is not possible, GPs should consider e-mental health options such as computerised cognitive behaviour therapy programs (see www.emhprac.org.au/directory/ for a directory of e-mental health resources), which are effective treatments for anxiety [47].

Finally, we are well aware that research often does not reflect real-world treatment settings and that practice guidelines frequently do not take into account the complexities of clinical practice. Future research should explore implementation barriers in more detail to determine how the guidelines for treating anxiety can be made more accessible and practical for GPs.

## Conclusions

Using the most comprehensive and current dataset on Australian GP activity available, this study found that anxiety is accounting for more of the GP workload, year on year. Over the period studied, referrals to psychologists tripled, prescription of SSRIs/SNRIs increased by 68%, and prescription of benzodiazepines decreased by almost 40%, suggesting GP management of anxiety has become more closely aligned with practice guidelines since 2006.

Systematic differences in management were found according to patient and GP characteristics, including high rates of management with benzodiazepines in older adults and patients with a Government health care concession card. Younger and female GPs, as well as those working in practices with other doctors, were less likely to manage anxiety with benzodiazepines and more likely to use SSRI/SNRI medications, referrals, and counselling. Further research is needed into GP treatment decision making for anxiety to understand these differences.

## Data Availability

The data that support the findings of this study are available from the University of Sydney’s BEACH data access committee, but restrictions apply to the availability of these data, which were used under license for the current study, and so are not publicly available. Data are however available from the data custodian (CH; christopher.harrison@sydney.edu.au) upon reasonable request and with permission of the University of Sydney’s Human Ethics Committee.

## Abbreviations

ASGS: Australian Statistical Geography Standard
ATC: Anatomical Therapeutic Chemical
BEACH: Bettering the Evaluation and Care of Health
CAE: Counselling, advice, or education
GP: General practitioner
HCC: Health care card
ICPC-2: International Classification of Primary Care Version 2
ISRAD: Index of Relative Socio-economic Advantage and Disadvantage
OR: Odds ratio
SEIFA: Socio-Economic Indexes for Areas
SNRI: Serotonin noradrenaline reuptake inhibitors
SSRI: Selective serotonin reuptake inhibitor
